# Aberrant expression of nuclear HDAC3 and cytoplasmic CDH1 predict a poor prognosis for patients with pancreatic cancer

**DOI:** 10.18632/oncotarget.7663

**Published:** 2016-02-24

**Authors:** Feng Jiao, Hai Hu, Ting Han, Meng Zhuo, Cuncun Yuan, Haiyan Yang, Lei Wang, Liwei Wang

**Affiliations:** ^1^ Department of Medical Oncology and Pancreatic Cancer Center, Shanghai General Hospital, Shanghai Jiao Tong University School of Medicine, Shanghai 201620, China; ^2^ Shanghai Key Laboratory of Pancreatic Diseases, Shanghai 201620, China; ^3^ Department of Pathology, Shanghai General Hospital, Shanghai Jiao Tong University School of Medicine, Shanghai 201620, China

**Keywords:** pancreatic cancer, histone deacetylases 3, CDH1, subcellular localization, prognosis

## Abstract

Previous studies showed that aberrant CDH1 or/and HDAC3 localization is essential for the progression of some human cancers. Here, we investigate the prognostic significance of aberrant CDH1 and HDAC3 localization in 84 pancreatic cancer patients. Our results show that increases in both membrane and cytoplasmic CDH1 correlate with lymph node metastasis (*P* = 0.026 and *P* < 0.001, respectively) and clinical stage (*P* = 0.020 and *P* < 0.001, respectively). Increased nuclear HDAC3 correlates with lymph node metastasis (*P* < 0.001) and advanced clinical stage (*P* < 0.001), but increased cytoplasmic HDAC3 does not (*P* > 0.05). Multivariate analysis showed that nuclear HDAC3 and cytoplasmic CDH1 (*P* = 0.001 and *P* = 0.010, respectively), as well as tumor differentiation (*P* = 0.009) are independent prognostic factors. Most importantly, patients with high co-expression of nuclear HDAC3 and cytoplasmic CDH1 had shorter survival times (*P* < 0.001), more frequent lymph node metastasis (*P* < 0.001), and advanced clinical stage (*P* < 0.001). Our studies provide convincing evidence that nuclear HDAC3 and cytoplasmic CDH1 have independent prognostic value in pancreatic cancer and provide novel targets for prognostic therapeutics.

## INTRODUCTION

Pancreatic cancer (PC) is one of the most aggressive and lethal malignancies, causing the deaths of an estimated 330,400 men and women worldwide in 2012 [1]. Total deaths due to PC are projected to increase dramatically, making it second leading cause of cancer-related deaths in the United States by 2030 [2]. Gemcitabine, the current standard first-line treatment, offers marginal symptom control and prolongation of life. Clinical trials aiming to improve the efficacy of gemcitabine have provided little improvement in survival outcomes [3]. New therapeutic strategies, including therapeutic antibodies or/and small molecule inhibitors, have been successful for a number of malignancies, but results obtained on PC treatments have so far been extremely frustrating [4]. A number of molecular mechanisms responsible for transformation and progression of PC have been identified, providing a set of potential pharmacological targets [5]. Among these is loss of adhesion between tumor cells caused by downregulation of CDH1 (also called E-cadherin) in response to genetic or epigenetic changes [6–8].

Histone acetylation is a dynamic epigenetic mechanism regulated by the histone acetyltransferases (HAT) and histone deacetylases (HDACs). HDAC3 (histone deacetylases 3), a member of class I HDACs, is overexpressed in the majority of carcinomas [9, 10], and is one of the most frequently upregulated genes in cancer [11]. Our previous study shows increased HDAC3 expression in PC [12]. HDAC3 could function as an oncogenic protein, promoting PC cell proliferation, migration, and invasion, as well as increasing drug resistance [12]. HDAC3 inversely correlates with CDH1 expression in ovarian carcinoma, and HDAC3 siRNA knock down in ovarian carcinoma cells reduced cell migration and increased CDH1 expression [13]. HDAC3 represses CDH1 through interactions with epithelial-mesenchymal transition (EMT) regulators including Snail and Twist1 [14].

This study uses high-throughput tissue microarray (TMA) and immunohistochemistry to investigate the expression and subcellular localization of CDH1 and HDAC3 in PC tissues. We analyze their association with clinicopathological factors, and address their possible value as prognostic indicators.

## RESULTS

### Expression of CDH1 and HDAC3 in PC tissues and adjacent normal tissues

**Figure 1:**
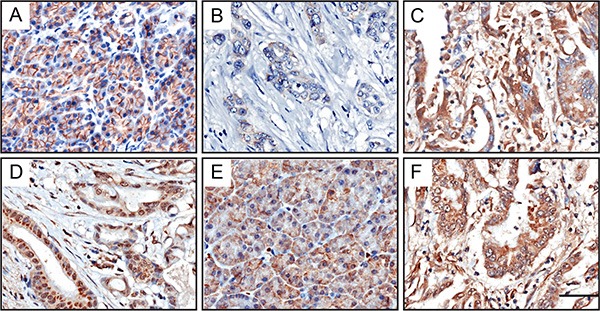
Immunohistochemical expression levels and localization of CDH1 and HDAC3 in PC tissues Strong membrane-associated CDH1 was observed in adjacent normal tissues (**A**). Low membrane CDH1 (**B**) and high cytoplasmic CDH1 (**C**) was found in tumor cells. Higher level of nuclear HDAC3 was observed in PC tissues (**D**), than in adjacent normal tissues (**E**). There was no difference in cytoplasmic HDAC3 expression between PC tissues and noncancerous samples (E, normal tissue; (**F**), tumor tissue). Scale bar, 50 μm.

Immunohistochemistry results are summarized in Tables 1 and 2. Strong membrane localization of CDH1 was observed in 85.7% (72/84) of normal tissues adjacent to PC (Figure 1A). In contrast, cell membrane expression of CDH1 was greatly reduced in PC tissues (Figure 1B), with high expression in 63.1% (53/84) of cases. Interestingly, higher cytoplasmic CDH1 expression was observed in PC samples (Figure 1C); 33.3% of tumor samples (28/84) but only 11.9% (10/84) of adjacent tissue samples displayed high cytoplasmic CDH1.

**Table 1:** Comparisons with CDH1 expression between PC and paired adjacent normal tissues (*n* = 84)

Tissue sample	No.of patients	Membrane CDH1 (*n*, %)		*P*-value	Cytoplasmic CDH1 (*n*, %)		*P*-value
		Low	High		Low	High	
Tumor	84	31 (36.9)	53 (63.1)	0.001*	56 (66.7)	28 (33.3)	0.001*
Adjacent normal	84	12 (14.3)	72 (85.7)		74 (88.1)	10 (11.9)	

**Table 2:** Comparisons with HDAC3 expression between PC and paired adjacent normal tissues (*n* = 84)

Tissue sample	No.of patients	Nuclear HDAC3 (*n*, %)		*P*-value	Cytoplasmic HDAC3 (*n*, %)		*P*-value
		Low	High		Low	High	
Tumor	84	38 (45.2)	46 (54.8)	< 0.001*	38 (45.2)	46 (54.8)	0.641
Adjacent normal	84	68 (81.0)	16 (19.0)		35 (41.7)	49 (58.3)	

HDAC3 was distributed in the cytoplasm and nucleus. As shown in Figure 1D, nuclear HDAC3 was highly expressed in 54.8% (46/84) of PC tissues. In contrast, HDAC3 was only seen in the nucleus of 19% (16/84) of noncancerous tissues (Figure 1E). There was no difference in cytoplasmic HDAC3 expression between PC tissues and noncancerous samples (54.8%, 46/84 vs. 58.3%, 49/84; Figure 1E, 1F).

### Correlations of CDH1 and HDAC3 expression in PC tissues

An inverse correlation was identified between low membrane expression of CDH1 and high nuclear HDAC3 expression (Spearman correlation coefficient *r* = −0.348, *P* = 0.001, Supplementary Table S1). High cytoplasmic CDH1 expression positively correlated with high nuclear HDAC3 expression (Spearman correlation coefficient *r* = 0.440, *P* < 0.001, Table 3). No correlations were found between cytoplasmic HDAC3 expression and CDH1 expression location (*P* > 0.05, Supplementary Tables S2, S3).

**Table 3:** Association between nuclear HDAC3 and cytoplasmic CDH1 expression

Tumor tissue sample	Nuclear HDAC3		Correlation coefficient	*P*-value
	Low	High		
Cytoplasmic CDH1 Low	34	22	0.440	< 0.001*
Cytoplasmic CDH1 High	4	24		

### Relationship of clinicopathological features with CDH1 and HDAC3 expression in PC patients

The relationships of CDH1 and HDAC3 expression levels with clinicopathological features of PC were evaluated by immunohistochemistry. As summarized in Table 4, CDH1 cell membrane expression correlated with lymph node metastasis (*P* = 0.026) and clinical stage (*P* = 0.020). High cytoplasmic CDH1 strongly correlated with lymph node metastasis (N classification, *P* < 0.001) and advanced clinical stage (*P* < 0.001). Neither cytoplasmic nor membrane CDH1 were associated with patients’ gender, age, tumor location, tumor size, tumor differentiation, invasion depth, distant metastasis, abdominal pain, jaundice or nervous invasion (*P* > 0.05).

**Table 4:** Correlation between the clinicopathologic characteristics and CDH1 expression (*n* = 84)

Clinicopathological parameters	No.of patients	Membrane CDH1 (*n*, %)			Cytoplasmic CDH1 (*n*, %)		
		Low	High	*P*-value	Low	High	*P*-value
Cases	84	31 (36.9)	53 (63.1)		56 (66.7)	28 (33.3)	
**Age (years)**							
≤ 60	39	17 (43.6)	22 (56.4)	0.237^a^	26 (66.7)	13 (33.3)	1.000^a^
> 60	45	14 (31.1)	31 (68.9)		30 (66.7)	15 (33.3)	
**Gender**							
Male	51	21 (41.2)	30 (58.8)	0.313^a^	34 (66.7)	17 (33.3)	1.000^a^
Female	33	10 (30.3)	23 (69.7)		22 (33.3)	11 (33.3)	
**Tumor location**							
Head, neck	56	24 (42.9)	32 (57.1)	0.110^a^	35 (62.5)	21 (37.5)	0.252^a^
Body, tail	28	7 (25.0)	21 (75.0)		21 (75.0)	7 (25.0)	
**Tumor size (cm)**							
≤ 3	25	9 (36.0)	16 (64.0)	0.911^a^	18 (72.0)	7 (28.0)	0.500^a^
> 3	59	22 (37.3)	37 (62.7)		38 (64.4)	21 (35.6)	
**Tumor differentiation**							
Well, moderate	57	21 (36.8)	36 (63.2)	0.986^a^	40 (70.2)	17 (29.8)	0.322^a^
Poor	27	10 (37.0)	17 (63.0)		16 (59.3)	11 (40.7)	
**Invasion depth**							
T1 + T2	71	27(38.0)	44(62.0)	0.618^a^	49 (69.0)	22 (31.0)	0.286^a^
T3 + T4	13	4(30.8)	9(69.2)		7 (53.8)	6 (46.2)	
**Lymph nodes metastasis**							
N0 (negative)	51	14 (27.5)	37 (72.5)	0.026^a^*	43 (84.3)	8 (15.7)	< 0.001^a^*
N1 (positive)	33	17 (51.5)	16 (48.5)		13 (39.4)	20 (60.6)	
**Distant metastasis**							
Absent	82	29 (35.4)	53 (64.6)	0.133^b^	56 (68.3)	26 (31.7)	0.108^b^
Present	2	2 (100)	0 (0)		0 (0)	2 (100)	
**Clinical stage**							
Early stages (≤ IIa)	49	13(26.5)	36(73.5)	0.020^a^*	43(87.8)	6(12.2)	< 0.001^a^*
Advanced stages (> IIa)	35	18(51.4)	17(48.6)		13(37.1)	22(62.9)	
**Abdominal pain**							
Absent	38	13 (34.2)	25 (65.8)	0.642^a^	22 (57.9)	16 (42.1)	0.121^a^
Present	46	18 (39.1)	28 (60.9)		34 (73.9)	12 (26.1)	
**Jaundice**							
Absent	69	23 (33.3)	46 (66.7)	0.146^a^	47 (68.1)	22 (31.9)	0.546^a^
Present	15	8 (53.3)	7 (46.7)		9 (60.0)	6 (40.0)	
**Nervous invasion**							
Negative	51	20 (39.2)	31 (60.8)	0.585^a^	33 (64.7)	18 (35.3)	0.636^a^
Positive	33	11 (33.3)	22 (66.7)		23 (69.7)	10 (30.3)	

a Chi-square test.

b Fisher's exact test.

* *P* < 0.05 indicates a significant association among the variables.

As summarized in Table 5, no correlations were observed between cytoplasmic levels of HDAC3 and patients’ clinicopathologic features. Nuclear HDAC3 staining correlated with lymph node metastasis (*P* < 0.001) and clinical stage (*P* < 0.001), but did not correlate with patient's gender, age, tumor location, tumor size, tumor differentiation, invasion depth, distant metastasis, abdominal pain, jaundice, or nervous invasion (*P* > 0.05).

**Table 5:** Correlation between the clinicopathologic characteristics and HDAC3 expression (*n* = 84)

Clinicopathological parameters	No.of patients	Nuclear HDAC3 (*n*, %)			Cytoplasmic HDAC3 (*n*, %)		
		Low	High	*P*-value	Low	High	*P*-value
Cases	84	38 (45.2)	46 (54.8)		38 (45.2)	46 (54.8)	
**Age (years)**							
≤ 60	39	19 (48.7)	20 (51.3)	0.551^a^	18 (46.2)	21 (53.8)	0.875^a^
> 60	45	19 (42.2)	26 (57.8)		20 (44.4)	25 (55.6)	
**Gender**							
Male	51	20 (39.2)	31 (60.8)	0.168^a^	25 (49.0)	26 (51.0)	0.387^a^
Female	33	18 (54.5)	15 (45.5)		13 (39.4)	20 (60.6)	
**Tumor location**							
Head, neck	56	23 (41.1)	33 (58.9)	0.278^a^	26 (46.4)	30 (53.6)	0.757^a^
Body, tail	28	15 (53.6)	13 (46.4)		12 (42.9)	16 (57.1)	
**Tumor size (cm)**							
≤ 3	25	10 (40.0)	15 (60.0)	0.530^a^	12 (48.0)	13 (52.0)	0.741^a^
> 3	59	28 (47.5)	31 (52.5)		26 (44.1)	33 (55.9)	
**Tumor differentiation**							
Well, moderate	57	27 (47.4)	30 (52.6)	0.569^a^	26 (45.6)	31 (54.4)	0.920^a^
Poor	27	11 (40.7)	16 (59.3)		12 (44.4)	15 (55.6)	
**Invasion depth**							
T1 + T2	71	35 (49.3)	36 (50.7)	0.081^a^	29 (40.8)	42 (59.2)	0.059^a^
T3 + T4	13	3 (23.1)	10 (76.9)		9 (69.2)	4 (30.8)	
**Lymph nodes metastasis**							
N0 (negative)	51	32 (62.7)	19 (37.3)	< 0.001^a^^*^	23 (45.1)	28 (54.9)	0.974^a^
N1 (positive)	33	6 (18.2)	27 (81.8)		15 (45.5)	18 (54.5)	
**Distant metastasis**							
Absent	82	38 (46.3)	44 (53.7)	0.499^b^	36 (43.9)	46 (56.1)	0.202^b^
Present	2	0 (0)	2 (100)		2 (100)	0 (0)	
**Clinical stage**							
Early stages (≤ IIa)	49	31 (63.3)	18 (36.7)	< 0.001^a^*	22 (44.9)	27 (55.1)	0.941^a^
Advanced stages (> IIa)	35	7 (20.0)	28 (80.0)		16 (45.7)	19 (54.3)	
**Abdominal pain**							
Absent	38	16 (42.1)	22 (57.9)	0.600^a^	19 (50.0)	19 (50.0)	0.425^a^
Present	46	22 (47.8)	24 (52.2)		19 (41.3)	27 (58.7)	
**Jaundice**							
Absent	69	33 (47.8)	36 (52.2)	0.307^a^	31 (44.9)	38 (55.1)	0.902^a^
Present	15	5 (33.3)	10 (66.7)		7 (46.7)	8 (53.3)	
**Nervous invasion**							
Negative	51	19 (37.3)	32 (62.7)	0.068^a^	26 (51.0)	25 (49.0)	0.189^a^
Positive	33	19 (57.6)	14 (42.4)		12 (36.4)	21 (63.6)	

a Chi-square test.

b Fisher's exact test.

* *P* < 0.05 indicates a significant association among the variables.

### Associations between CDH1 and HDAC3 expression and survival

**Figure 2:**
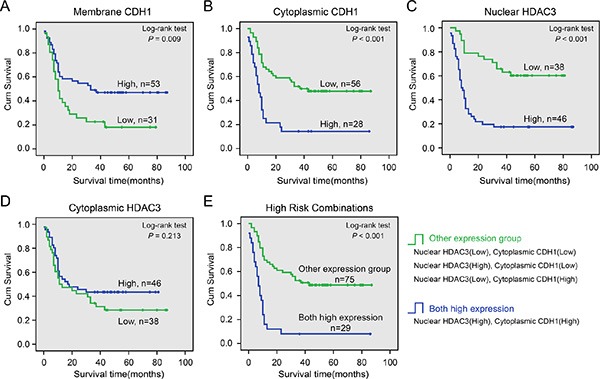
Cumulative kaplan-meier overall survival curves of 84 PC patients segmented by CDH1 (A), membrane CDH1; (B), cytoplasmic CDH1), HDAC3 (C), nuclear HDAC3; (D), cytoplasmic HDAC3), and high-risk combination group (cytoplasmic CDH1 and nuclear HDAC3 combinations) (E) *P*-values were calculated by the log-rank test.

Kaplan-Meier analysis and log-rank test were used to investigate the prognostic value of CDH1 and HDAC3 expression and classic clinicopathologic characteristics on patient survival. In univariate analysis, both membrane and cytoplasmic CDH1 expression, as well as nuclear HDAC3, were closely associated with overall survival (OS) of PC patients (*P* = 0.012, *P* < 0.001, and *P* < 0.001, respectively; Table 6), with Spearman correlation coefficients of 0.240, −0.435, and −0.530 (Supplementary Table S4), respectively. The log-rank test results showed that the aberrant expression levels of these proteins correlated strongly with poorer survival in PC patients (*P* < 0.001; Figure 2). As shown in Table 7, the cumulative 1-year survival rate was 58% in the high membrane CDH1 group (95% confidence interval [CI], 0.443–0.717), whereas it was only 32% (95% CI, 0.163–0.477) in the low expression group (Figure 2A). The cumulative 1-year survival rate was 63% (95% CI, 0.512–0.748) in the low cytoplasmic CDH1 group, whereas it was only 21% (95% CI, 0.053–0.367) in the high-expression group (Figure 2B). The 1-year survival rate was 79% in the low nuclear HDAC3 group (95% CI, 0.653–0.927), whereas it was only 24% (95% CI, 0.122–0.358) in the high staining group (Figure 2C). There was no difference in survival time associated with cytoplasmic HDAC3 expression (low vs. high, 47% (95% CI, 0.313–0.627) vs. 50% (95% CI, 0.363–0.637); Figure 2D).

**Table 6:** Summary of univariate and multivariate Cox regression analysis of overall survival duration in all PCs

Clinicopathological parameters	Univariate analysis			Multivariate analysis		
	HR	95% CI	*P*-value	HR	95% CI	*P*-value
**Membrane CDH1**						
Low	1					
High	0.500	0.290–0.861	0.012*			
**Cytoplasmic CDH1**						
Low	1			1		
High	2.996	1.725–5.204	< 0.001*	2.204	1.210–4.012	0.010*
**Nuclear HDAC3**						
Low	1			1		
High	4.020	2.182–7.405	< 0.001*	3.033	1.572–5.852	0.001*
**Cytoplasmic HDAC3**						
Low	1					
High	0.716	0.418–1.227	0.224			
**Age (years)**						
≤ 60	1					
> 60	0.956	0.558–1.639	0.870			
**Gender**						
Male	1					
Female	0.531	0.295–0.957	0.035*			
**Tumor location**						
Head, neck	1					
Body, tail	1.189	0.678–2.085	0.546			
**Tumor size(cm)**						
≤ 3	1					
> 3	0.797	0.451–1.409	0.436			
**Tumor differentiation**						
Well, moderate	1			1		
Poor	2.077	1.192–3.620	0.010*	2.119	1.210–3.711	0.009*
**Invasion depth**						
T1 + T2	1					
T3 + T4	0.983	0.463–2.088	0.965			
**Lymph nodes metastasis**						
N0(negative)	1					
N1(positive)	2.060	1.196–3.546	0.009*			
**Distant metastasis**						
Absent	1					
Present	2.372	0.574–9.798	0.233			
**Clinical stage**						
Early stages (≤ IIa)	1					
Advanced stages (> IIa)	2.230	1.294–3.845	0.004*			
**Abdominal pain**						
Absent	1					
Present	0.913	0.531–1.569	0.742			
**Jaundice**						
Absent	1					
Present	0.976	0.476–2.000	0.947			
**Nervous invasion**						
Negative	1					
Positive	1.168	0.678–2.012	0.576			

HR hazard ratio, 95% CI 95% confidence interval.

**Table 7:** Comparisons with cumulative 1-year survival rate between different groups

Variables	Cumulative 1-year survival rate	95% CI
**Membrane CDH1**		
Low	32%	0.163–0.477
High	58%	0.443–0.717
**Cytoplasmic CDH1**		
Low	63%	0.512–0.748
High	21%	0.053–0.367
**Nuclear HDAC3**		
Low	79%	0.653–0.927
High	24%	0.122–0.358
**Cytoplasmic HDAC3**		
Low	47%	0.313–0.627
High	50%	0.363–0.637
**High risk combinations**		
Both high expression	12%	0.002–0.238
Other expression group	64%	0.522–0.758

95% CI, 95% confidence interval.

Univariate analysis also indicated that gender, tumor differentiation, lymph node metastasis, and clinical stage correlated with patient survival (*P* = 0.035, *P* = 0.010, *P* = 0.009, and *P* = 0.004, respectively). Multivariate analysis shows that cytoplasmic CDH1 expression, nuclear HDAC3 expression, and tumor differentiation were independent prognostic factors for PC patients (Table 6). Membrane CDH1 expression, gender, lymph node metastasis, and clinical stage were not associated with survival (Table 6). To further investigate the association of survival time with cytoplasmic CDH1 and nuclear HDAC3 expression, a final concomitant model was constructed. As shown in Figure 2E, the log-rank test showed that high co-expression of these two proteins correlated with shorter survival time of PC patients (*P* < 0.001). The cumulative proportion of 1-year survival was only 12% (95% CI, 0.002–0.238) in the high co-expression group and 64% (95% CI, 0.522–0.758) in other combination groups (Table 7). Moreover, Spearman correlation analysis revealed a positive correlation between the high co-expression group and lymph nodes metastasis, clinical stage (*r* = 0.436 and *r* = 0.506, respectively, Supplementary Table S5).

## DISCUSSION

Cellular functions are dictated by protein activity and content. There are numerous strategies to regulate proteins varying from modulating gene expression to post-translational modifications to control of protein localization [15]. Numerous studies demonstrate functionally relevant subcellular translocation of specific individual proteins [16]. For example, β-catenin is found at multiple subcellular localizations, including at cell junctions, where it stabilizes cell-cell contacts; in the cytoplasm, where β-catenin levels are controlled by protein stability regulating processes; and in the nucleus, where β-catenin is involved in transcriptional regulation and chromatin interactions [17, 18]. Moreover, β-catenin nuclear import and accumulation drives tumor formation and correlates with clinical tumor grade [19]. Another example is BRCA1, whose prognostic significance varies with its subcellular distribution. Nuclear detection of the protein is associated with a worse prognosis, while cytoplasmic localization predicts lower probability of recurrence due to fewer lymph node metastases [20].

Dysfunction of the CDH1-mediated cell adhesion system plays an important role in pancreatic tumor progression to invasive, metastatic carcinoma [21, 22]. Epigenetic modifications contribute to loss of CDH1 expression [23, 24]. Yao R *et al.* [25] found that HDAC3 binds the CDH1 promoter, resulting in reduced local histone acetylation and CDH1 transcriptional repression [25]. We previously revealed that HDAC3 is overexpressed in PC tissue, and increased HDAC3 can promote malignant tumor phenotypes [12]. Moreover, Hayashi A *et al.* [13] found that HDAC3 was inversely correlated with CDH1 expression in ovarian carcinoma. In this study, we determined the expression pattern of CDH1 and HDAC3 proteins in PC tissues, and the clinicopathological and prognostic value of those subcellular localizations.

High-throughput TMA was employed to perform our research. First, we found that CDH1 was predominantly found on the cell membrane and in the cytoplasm, while HDAC3 localized to cell nucleus and cytoplasm. Further analysis revealed that the cell membrane CDH1 was greatly reduced in PC tissues compared to noncancerous epithelia, whereas nuclear HDAC3 was abnormally upregulated. Furthermore, there was an inverse association between these two proteins in PC tissues, consistent with recent reports on ovarian carcinoma [13].

It is worth noting that abnormal cytoplasmic CDH1 in PC tissues, and higher cytoplasmic CDH1 expression were associated with more aggressive tumor-associated variables, including lymph node metastasis and advanced clinical stage. Moreover, PC patients with high cytoplasmic CDH1 expression had shorter OS than the low-expression group. In contrast, reduced membrane CDH1 correlated with lymph node metastasis, advanced clinical stage, and shorter survival time. Multivariate analyses demonstrate that cytoplasmic but not membrane CDH1 expression was an independent prognostic factor for PC. Previously, Deeb G *et al.* [26] found that cytoplasmic staining of CDH1 in lung cancer tissues correlates with shorter patient survival. Ito K *et al.* [27] revealed that CDH1 cytoplasmic staining may be due to CDH1 proteolytic cleavage by a membrane-bound metalloprotease, yielding a soluble form. Although nuclear staining of CDH1 protein has been associated with skin Merkel cell carcinomas [28], we did not observe nuclear CDH1 in our PC patient cohort. Taken together, cytoplasmic CDH1 expression appears to represent altered protein localization related to PC tumorigenicity.

HDAC3 is the only class I HDAC found in the nucleus, cytoplasm, and plasma membrane [29, 30]. Previous studies focused on its function as an epigenetic modifier, repressing transcription through histone deacetylation [10, 31, 32]. Few studies have investigated the prognostic role of altered HDAC3 localization in PC. In this study, we found HDAC3 in the cytoplasm and nucleus of tumor cells, but not on the plasma membrane. Higher nuclear HDAC3 expression was observed in PC relative to adjacent normal tissues, while cytoplasmic expression of HDAC3 was indistinguishable. Cytoplasmic staining of HDAC3 was not associated with any clinicopathologic features or survival in PC patients. In contrast, increased nuclear HDAC3 expression was strongly associated with N classification and advanced clinical stage. For example, nuclear HDAC3 expression was detected in 80.0% of patients with high tumor grade (> IIa), but only 36.7% in the low tumor grade group (≤ IIa), suggesting that nuclear HDAC3 plays an important role in tumor progression in PC patients. Univariate analysis showed that nuclear HDAC3 in PC was associated with patients’ OS. Higher nuclear HDAC3 correlates with worse prognosis. Furthermore, according to multivariate analysis, overexpression of nuclear HDAC3 has independent prognostic significance for PC. It is of particular note that high nuclear HDAC3 expression was positively associated with increased cytoplasmic CDH1. High co-expression of these two proteins correlated with shorter patient survival, with a cumulative 1-year survival of 12% (95% CI, 0.002–0.238) compared to that of 64% (95% CI, 0.522–0.758) in other expression levels group. Escaffit F *et al.* [33] reported that nuclear localization of HDAC3 decreases the efficiency of apoptosis induction, and HDAC3 cytoplasmic relocalization is important for the apoptotic process.

We speculate that first, pancreatic tumor cells may have escaped apoptosis, at least in part, through HDAC3 overexpression in cell nucleus. Secondly, high concentrations of nuclear HDAC3 may directly inhibit CDH1 promoters, leading to reduced CDH1 cell membrane expression. Additionally, nuclear HDAC3 expression may upregulate membrane-bound metalloprotease expression through epigenetic modification of the associated target gene, leading to increased cytoplasmic CDH1. Together, our findings strongly indicate that nuclear HDAC3 upregulation is crucial for the aggressive behaviors and worse prognosis of PC patients, which suggest that HDAC3 may be an effective therapeutic target. Unfortunately, clinical data for HDAC inhibitors (HDACIs) are inadequate, because few studies have included patients with PC and few PC patients entered the HDACIs phase II/III trials that did [34]. More high quality clinical trials recruiting candidates with PC are required to determine the efficacy of these therapies. Selective HDACIs, potentially targeting HDAC3, may yield more potent efficacy and fewer side effects than pan-HDACIs.

In summary, these data strongly suggest the importance of nuclear HDAC3 and cytoplasmic CDH1 in the progression and clinical outcome of human PC. These markers provide strong candidates for targeted therapy of PC patients. Larger prospective studies could further validate these findings.

## MATERIALS AND METHODS

### Patients and tissue samples

This study was approved by the Ethics and Research Committees of Shanghai General Hospital, Shanghai Jiao Tong University School of Medicine, and was conducted in accordance with the Declaration of Helsinki Principles. TMAs containing 90 PC tissues and corresponding non-tumor tissues were purchased from ShGnghGi Outdo Biotech Company (China). The TMAs contained well-documented clinicopathological information, including patients’ age, sex, tumor size and location, tumor differentiation, invasion depth, lymph node metastasis, distant metastasis, clinical stage, abdominal pain, jaundice, nervous invasion, and follow-up data (ended in December, 2011). Six patients were excluded due to lack of completed clinical and follow-up data. In total, 84 patients were included, 51 males and 33 females, with a median age of 62 years old (ranging from 38 to 85 years old). The overall survival time ranged from 0 to 87 months, with a median of 15 months. Detailed information can be found in Table 8.

**Table 8:** Detailed clinical information of patients with PC

Characteristics	Categories	Number
Overall survival median (range, months)		15 (0–87)
Age median (range, years)		62 (38–85)
Tumor location	Head, neck	56
	Body, tail	28
Tumor size (cm)	≤ 3	25
	> 3	59
Tumor differentiation	Well, moderate	57
	Poor	27
Invasion depth	T1 + T2	71
	T3 + T4	13
Lymph nodes metastasis	N0 (negative)	51
	N1 (positive)	33
Distant metastasis	Absent	82
	Present	2
Clinical stage	Early stages (≤ IIa)	49
	Advanced stages (> IIa)	35
Abdominal pain	Absent	38
	Present	46
Jaundice	Absent	69
	Present	15
Nervous invasion	Negative	51
	Positive	33

### Immunohistochemistry

Immunohistochemistry was performed based on the standard streptavidin-peroxidase (S-P) method (Zymed, San Francisco, CA). After deparaffinization and rehydration, TMA sections were subjected to high pressure for antigen retrieval for 5 minutes. Endogenous peroxidase activity was blocked using 100 μL of peroxidase block for 10 min. The slides were subsequently incubated overnight at 4°C with primary antibodies as follows: CDH1 (dilution 1:300, BD Biosciences), HDAC3 (dilution 1:500, Abcam). After washing in 1× phosphate buffered saline (PBS), the sections were incubated with biotinylated secondary antibodies (Zymed, San Francisco, CA) for 30 min at room temperature, followed by incubation with streptavidin horseradish peroxidase complex. Finally, sections were incubated with DAB for 2 min. Positive controls were used in each experiment following supplier's instructions. Negative controls applying appropriate IgG to replace primary antibody were also run in each experiment (Supplementary Figure 1A, 1B).

### Scoring of immunohistochemistry

A double-blind method, carried out independently by two investigators without access to the patients’ clinical and pathological features, was used to analyze immunohistochemistry results. Five visual fields from different areas of each specimen were chosen at random for the immunohistochemistry evaluation. HDAC3 and CDH1 expression was scored according to staining intensity and the percentage of positive cells as previously described [35]. The percentage of positive cells was scored as follows: 0% (0), 1%–10% (1), 11%–50% (2) and 51%–100% (3). Staining intensity was scored as follows: no staining (0), week (1), moderate (2), and strong (3). Comprehensive score = staining percentage × intensity. CDH1 or HDAC3 expression was classified as follows: < 6 low expression, ≥ 6 high expression.

### Statistical analysis

All statistical analyses were carried out using the SPSS 13.0 software. The χ^2^ test and Fisher's exact test were used to analyze the correlation between the clinicopathologic characteristics and CDH1 and HDAC3 expression as appropriate. Overall survival (OS) was defined as the interval from date of diagnosis until death from any cause. Data were censored for living patients and patients lost between follow-ups. The OS was estimated using the Kaplan-Meier method and compared using the log-rank test. Significant variables were further analyzed by multivariate analysis to test for independent prognosis. Bivariate correlations between variable factors were calculated by Spearman rank correlation coefficients. *P*-values < 0.05 were considered statistically significant.

## SUPPLEMENTARY MATERIALS FIGURE AND TABLES


