# Supplementary material for: Aberrant expression of nuclear HDAC3 and cytoplasmic CDH1 predict a poor prognosis for patients with pancreatic cancer
Source: Oncotarget. 2016 Feb 24;7(13):16505–16. doi: 10.18632/oncotarget.7663 (PMC4941331; doi:10.18632/oncotarget.7663)
Supplement: Supplementary file 1 [file oncotarget-07-16505-s001.pdf]

Aberrant expression of nuclear HDAC3 and cytoplasmic CDH1 predict a poor prognosis for patients with pancreatic cancer

Supplementary Materials

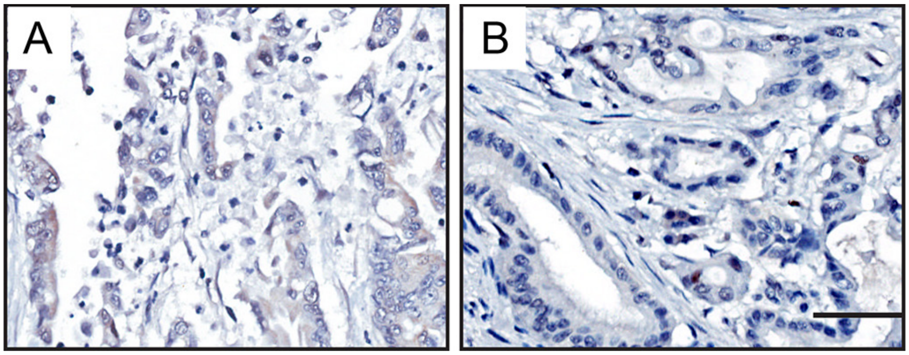

Supplementary Figure S1: Immunohistochemical staining of negative control for CDH1 (A) and HDAC3 (B) in PC tissues. Scale bar, 50 μm.

Supplementary Table S1: Association between nuclear HDAC3 and cell membrane CDH1 expression

Tumor tissue sample	Nuclear HDAC3		Correlation coefficient	*P*-value
	Low	High		
Membrane CDH1 Low	7	24	−0.348	0.001*
Membrane CDH1 High	31	22		

Supplementary Table S2: Association between cytoplasmic HDAC3 and cell membrane CDH1 expression

Tumor tissue sample	Cytoplasmic HDAC3		Correlation coefficient	*P*-value
	Low	High		
Membrane CDH1 Low	14	17	−0.001	0.991
Membrane CDH1 High	24	29		

Supplementary Table S3: Association between cytoplasmic HDAC3 and cytoplasmic CDH1 expression

Tumor tissue sample	Cytoplasmic HDAC3		Correlation coefficient	*P*-value
	Low	High		
Cytoplasmic CDH1Low	26	30	0.034	0.760
Cytoplasmic CDH1 High	12	16		

Supplementary Table S4: Spearman correlation analysis between CDH1 and HDAC3 and cumulative survival

Variables	Cumulative survival	
	Spearman correlation	*P*-value
Membrane CDH1	0.240	0.028
Cytoplasmic CDH1	−0.435	< 0.001*
Nuclear HDAC3	−0.530	< 0.001*
Cytoplasmic HDAC3	0.088	0.424

*$P < 0.05$ statistically significant.

Supplementary Table S5: Spearman correlation analysis between high co-expression group and clinical stage and lymph node metastasis

Variables	Co-expression group	
	Spearman correlation	*P*-value
Clinical stage	0.506	< 0.001*
Lymph nodes metastasis	0.436	< 0.001*
